# B Cells on the Stage of Inflammation in Juvenile Idiopathic Arthritis: Leading or Supporting Actors in Disease Pathogenesis?

**DOI:** 10.3389/fmed.2022.851532

**Published:** 2022-04-04

**Authors:** Rita A. Moura, João Eurico Fonseca

**Affiliations:** ^1^Instituto de Medicina Molecular João Lobo Antunes, Faculdade de Medicina, Universidade de Lisboa, Lisbon, Portugal; ^2^Rheumatology Department, Hospital de Santa Maria, Centro Hospitalar Universitário Lisboa Norte (CHULN), Lisbon Academic Medical Centre, Lisbon, Portugal

**Keywords:** juvenile idiopathic arthritis (JIA), B cells, autoantibodies, inflammation, immunopathogenesis

## Abstract

Juvenile idiopathic arthritis (JIA) is a term that collectively refers to a group of chronic childhood arthritides, which together constitute the most common rheumatic condition in children. The International League of Associations for Rheumatology (ILAR) criteria define seven categories of JIA: oligoarticular, polyarticular rheumatoid factor (RF) negative (RF-), polyarticular RF positive (RF+), systemic, enthesitis-related arthritis, psoriatic arthritis, and undifferentiated arthritis. The ILAR classification includes persistent and extended oligoarthritis as subcategories of oligoarticular JIA, but not as distinct categories. JIA is characterized by a chronic inflammatory process affecting the synovia that begins before the age of 16 and persists at least 6 weeks. If not treated, JIA can cause significant disability and loss of quality of life. Treatment of JIA is adjusted according to the severity of the disease as combinations of non-steroidal anti-inflammatory drugs (NSAIDs), synthetic and/ or biological disease modifying anti-rheumatic drugs (DMARDs). Although the disease etiology is unknown, disturbances in innate and adaptive immune responses have been implicated in JIA development. B cells may have important roles in JIA pathogenesis through autoantibody production, antigen presentation, cytokine release and/ or T cell activation. The study of B cells has not been extensively explored in JIA, but evidence from the literature suggests that B cells might have indeed a relevant role in JIA pathophysiology. The detection of autoantibodies such as antinuclear antibodies (ANA), RF and anti-citrullinated protein antibodies (ACPA) in JIA patients supports a breakdown in B cell tolerance. Furthermore, alterations in B cell subpopulations have been documented in peripheral blood and synovial fluid from JIA patients. In fact, altered B cell homeostasis, B cell differentiation and B cell hyperactivity have been described in JIA. Of note, B cell depletion therapy with rituximab has been shown to be an effective and well-tolerated treatment in children with JIA, which further supports B cell intervention in disease development.

## Introduction

**Figure 1:**
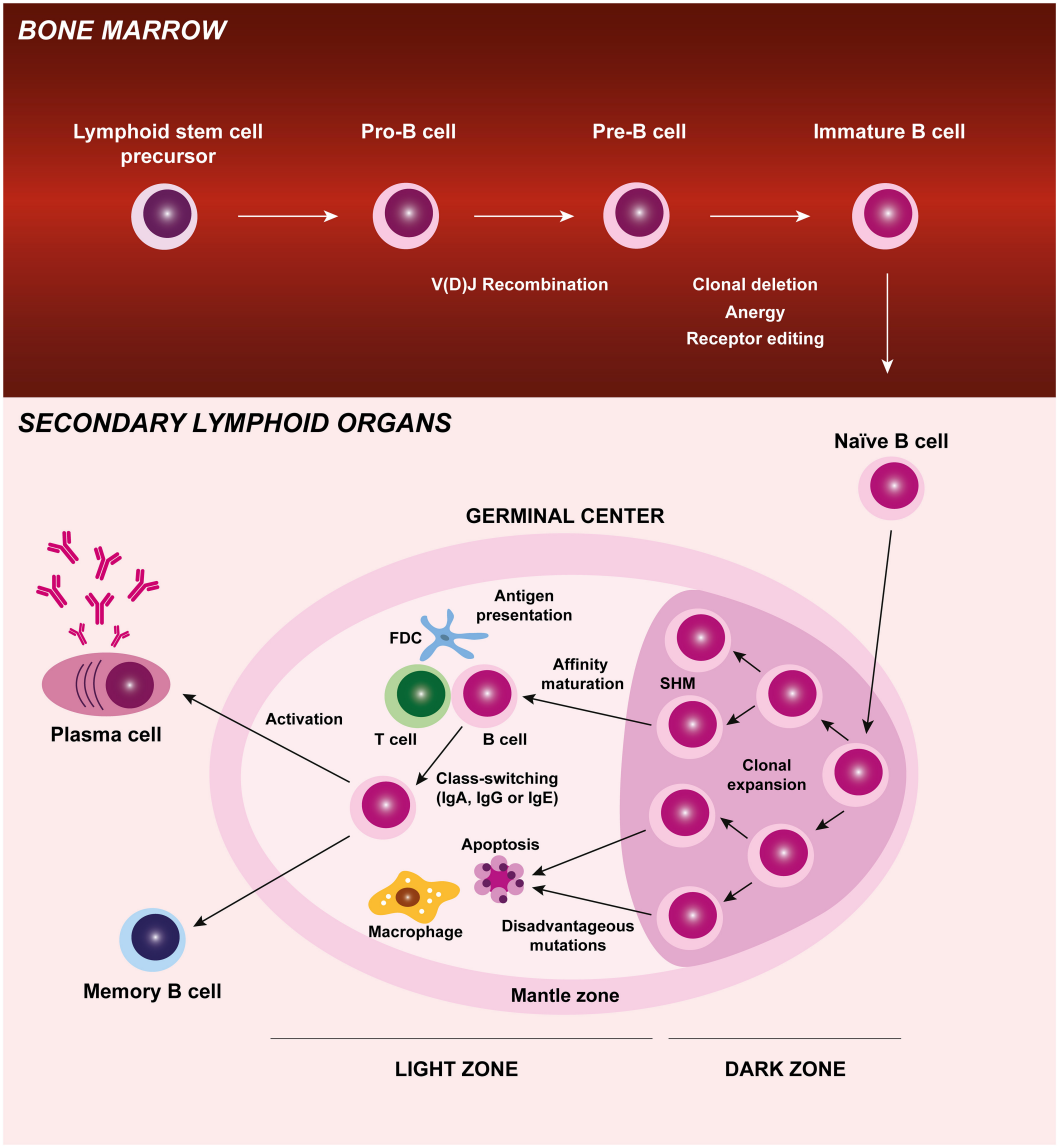
B cell origin and development. B cells originate from a lymphoid stem cell in the bone marrow and proceed through several maturation stages, during which V(D)J recombination and central tolerance mechanisms (clonal deletion, anergy, and receptor editing) occur. B cells leave the bone marrow and are released in the periphery as immature cells. These antigen-naïve B cells circulate through the blood and lymphatic systems to secondary lymphoid organs (lymph nodes, spleen, and Peyer's Patches) where, upon activation and exposure to antigen, they proliferate and differentiate into memory B cells or antibody-producing plasma cells. During B cell differentiation process at germinal centers, B cells are clonally expanded and go through somatic hypermutation, affinity maturation, and class-switching. Autoreactive B cells that may develop during B cell differentiation process and recognize self-antigens are eliminated by apoptosis. FDC, follicular dendritic cell; Ig, immunoglobulin; SHM, somatic hypermutation.

B cells have several important roles in autoimmunity such as autoantibody production, antigen presentation, cytokine release, and T cell activation. B cell development originates in the bone marrow, where these cells undergo different maturation stages and critical checkpoint mechanisms to ensure tolerance and are released in the periphery as immature cells. These antigen-naïve B cells circulate through the blood and lymphatic systems to secondary lymphoid organs where, upon activation and exposure to antigen, they proliferate and differentiate into memory B cells or antibody-producing plasma cells. During B cell differentiation process at germinal centers, B cells are clonally expanded and go through somatic hypermutation, affinity maturation and class or isotype switching (Figure 1) (1). Defects in central tolerance mechanisms (clonal deletion, anergy, and/ or receptor editing) occurring in the bone marrow and/ or during peripheral tolerance can contribute to the development of autoreactive B cells and autoimmune diseases (2–4). Juvenile idiopathic arthritis (JIA) is the most common rheumatic disorder in children. Disturbances in both innate and adaptive immune systems have been described in JIA patients. The study of B cells has not been extensively explored in JIA, but evidence from the literature suggests that B cells might have a relevant role in JIA pathogenesis (5–7). Notably, the detection of autoantibodies such as antinuclear antibodies (ANA), rheumatoid factor (RF), and anti-citrullinated protein antibodies (ACPA) in JIA patients supports a breakdown in B cell tolerance (8). Furthermore, it has been shown that JIA patients have increased rates of secondary V(D)J recombination (normally restricted to early B-cell precursors in the bone marrow) in peripheral blood B cells, with a skewed kappa (κ):lambda (λ) light chain usage (9–11). These data suggest that mature peripheral blood B cells of JIA patients have the potential to perform receptor revision outside the bone marrow and, therefore, promote autoimmunity (9–11). In addition, altered B cell homeostasis, B cell differentiation, and B cell hyperactivity have been described in JIA, which further supports B cell intervention in disease development (12–19).

## Juvenile Idiopathic Arthritis: Definition, Classification and Disease Categories

Juvenile idiopathic arthritis (JIA) is a term used to classify a group of heterogeneous chronic childhood inflammatory arthritides of unknown etiology, which together constitute the most common rheumatic condition in children (20). JIA is characterized by a chronic inflammatory process affecting the synovia that begins before the age of 16 and persists at least 6 weeks. Nonetheless, in some children, JIA can be a lifelong condition. JIA affects not only joints, but also extra-articular structures, including eyes, skin, and internal organs and, if not treated, can lead to serious disability and loss of quality of life (21, 22). The International League of Associations for Rheumatology (ILAR) criteria define seven categories of JIA: oligoarticular, polyarticular RF negative (RF-), polyarticular RF positive (RF+), systemic, enthesitis-related arthritis, psoriatic arthritis, and undifferentiated arthritis (23). The ILAR classification includes persistent and extended oligoarthritis as subcategories of oligoarticular JIA, but not as distinct categories. JIA initial classification is determined according to the clinical features presented during the first 6 months of disease course, such as the number of affected joints, severity of disease, and presence or absence of inflammation in other parts of the body. The onset of new clinical features during the course of the disease determines the final disease subtype.

### Oligoarticular

Oligoarticular JIA (oJIA) is the most common JIA subtype. It is defined as asymmetric arthritis affecting up to four joints, mainly large joints such as the knee, ankle, wrist, and/ or elbow, during the first 6 months after disease onset (23). oJIA can be subcategorized in two types of arthritis: *persistent oJIA* (no more than four joints are affected during the course of the disease) and *extended oJIA* (more than four joints are affected after the first 6 months of disease). oJIA usually begins before 4 years of age and female gender is predominantly affected (24). ANA are present in up to 60–80% of patients with oJIA. Importantly, oJIA is associated with a high risk of uveitis (24, 25), particularly in ANA positive (ANA+) patients (26–28).

### Polyarticular

Polyarticular JIA (pJIA) is the second most common JIA subtype, defined as arthritis in which 5 or more joints are affected during the first 6 months of the disease. There are two forms of pJIA: polyarticular RF negative (pJIA RF-) and polyarticular RF positive (pJIA RF+) (23). Female gender is predominant in both types of pJIA.

#### Polyarticular Rheumatoid Factor Negative

Patients with pJIA RF- have mostly asymmetric arthritis of small and large joints, and disease onset usually occurs between 2 and 12 years old. ANA can be detected in up to 40% of pJIA patients and uveitis can be present in up to 10% of cases (23–25).

#### Polyarticular Rheumatoid Factor Positive

Patients with pJIA RF+ have symmetric arthritis mainly of small joints (metacarpophalangeal joints and wrists) and disease onset is more frequent in late childhood or adolescence. Arthritis development is associated to a more erosive and aggressive disease progression (23–25).

### Systemic

Systemic JIA (sJIA) is a disease subtype defined by the presence of arthritis in one or more joints and concomitant systemic manifestations that include fever persisting for more than 2 weeks, generalized lymphadenopathy, rash, hepatosplenomegaly, and/ or serositis (23). Disease onset may occur at any time during childhood and both female and male genders are equally affected. Autoantibodies are usually absent (25, 29). A dysregulation of the innate immune system has been associated to the systemic inflammation present in sJIA, suggesting that this JIA subtype may rather be part of the spectrum of autoinflammatory disorders (29–33). Nevertheless, alterations in adaptive immunity have also been described in sJIA (34–38).

### Enthesitis-Related Arthritis

Enthesitis-related arthritis (ERA) is a subtype of JIA that affects the joints of the lower limbs (hip, knee, ankle, and foot) in association with enthesitis. In addition, axial involvement and arthritis of the sacroiliac joints and upper limbs, particularly the shoulders, can also occur (23). ERA is more frequent in male gender. Disease onset usually occurs in late childhood or adolescence. Acute anterior uveitis and gut inflammation can also be present in ERA patients. The diagnosis of this JIA subtype is strongly associated to the major histocompatibility complex (MHC) class I antigen human leukocyte antigen (HLA)-B27 (25).

### Psoriatic Arthritis

Juvenile psoriatic arthritis (JPsA) is characterized by an asymmetric arthritis of small and large joints and the presence either of a psoriatic rash or, in the absence of rash, at least two of the following criteria: first-degree relative with psoriasis, nail pitting or onycholysis, and dactylitis (23). Clinical symptoms may also include uveitis. JPsA is composed of two subgroups, differentiated by age at onset. Children with early-onset JPsA (<6 years old) are predominantly female, ANA+, more predisposed to uveitis, with arthritis of the wrists and small joints of the hands and feet. In contrast, children with later-onset JPsA are more associated to male gender, axial disease, enthesitis, and HLA-B27 positivity (39, 40).

### Undifferentiated Arthritis

Undifferentiated juvenile idiopathic arthritis includes patients who do not fulfill the criteria for any JIA category above described, or who meet the criteria for more than one (23, 41).

The main clinical features of all JIA categories are summarized in Table 1. Notably, the complexity and heterogeneity of JIA diagnosis is still controversial and subject to new classification proposals (42, 43).

**Table 1:** Clinical features of juvenile idiopathic arthritis.

	**JIA category**					
	**Oligoarticular**	**Polyarticular RF−**	**Polyarticular RF+**	**ERA**	**JPsA**	**Systemic**
Clinical prevalence	50–60% of all JIA	10–30% of all JIA	5–10% of all JIA	10–15% of all JIA	5–6% of all JIA	10–20% of all JIA
Gender predominance	Female	Female	Female	Male	Female (early-onset) Male (later-onset)	Equal
Pattern of arthritis	≤ 4 joints affected; Mainly large joints; Asymmetric	≥5 joints affected; Small and large joints; Symmetric or asymmetric	≥5 joints affected; Mainly small joints; Aggressive symmetric polyarthritis; Erosive	Lower limb joints more commonly affected; Axial involvement; Sacroiliac joints; Upper limbs	Small and large joints; Asymmetric	≥1 joint affected; Mostly wrists, knees, ankles or asymptomatic temporomandibular arthritis
Systemic manifestations	Uveitis	Uveitis	Rheumatoid nodules; Uveitis	Acute anterior uveitis; Enthesitis; Gut inflammation	Psoriasis; Dactylitis; Onycholysis; Nail pitting; Uveitis	Fever; Generalized lymphadenopathy; Rash; Serositis; Hepatosplenomegaly; MAS
Autoantibodies	ANA	ANA, ACPA	ANA, RF, ACPA	ANA (some cases)	ANA (early-onset)	Usually absent
Disease biomarkers	60–80% ANA+	40% ANA+	RF+, ACPA+, 40% ANA+	45–85% HLA-B27+	50% ANA+	Increased levels of CRP, Ferritin, Leukocytosis, Thrombocytosis

*ACPA, anti-citrullinated protein antibodies; ANA, antinuclear antibodies; CRP, C-reactive protein; ERA, enthesitis-related arthritis; HLA-B27, human leukocyte antigen-B27; JIA, juvenile idiopathic arthritis; JPsA, juvenile psoriatic arthritis; MAS, macrophage activation syndrome; RF, rheumatoid factor*.

## Etiology and Risk Factors of Juvenile Idiopathic Arthritis

The cause of JIA is unknown. Nevertheless, JIA has been established as an autoimmune disorder in which genetic susceptibility and environmental factors are associated to disease development. JIA might be initially triggered by the exposure to environmental factors in children with a genetic predisposition to synovial inflammation. Infections, vaccines, antibiotics, vitamin D deficiency, stress, and trauma have been proposed as environmental risk factors for JIA progression (25, 44, 45). In fact, it has been reported that infectious viruses (Epstein-Barr virus, Parvovirus B, Rubivirus, and Hepatitis B virus) and bacteria (*Salmonella* spp., *Shigella* spp., *Campylobacter* spp., *Streptococcus pyogenes, Bartonella henselae, Mycoplasma pneumoniae*, and *Chlamydophila pneumonia*) may act as triggering agents of JIA (46). Furthermore, disturbances in the gut microbiome have been shown to increase the risk of JIA development (47–49). Additionally, evidence from the literature suggests that maternal smoking during pregnancy can also be a risk factor for JIA (44, 50). Genetic predisposition to JIA is mainly due to human leukocyte antigen (HLA) class II molecules, although HLA class I molecules and non-HLA genes have also been implicated, depending on the disease category (24, 25, 29, 51–57).

## Immunopathogenesis of Juvenile Idiopathic Arthritis

**Figure 2:**
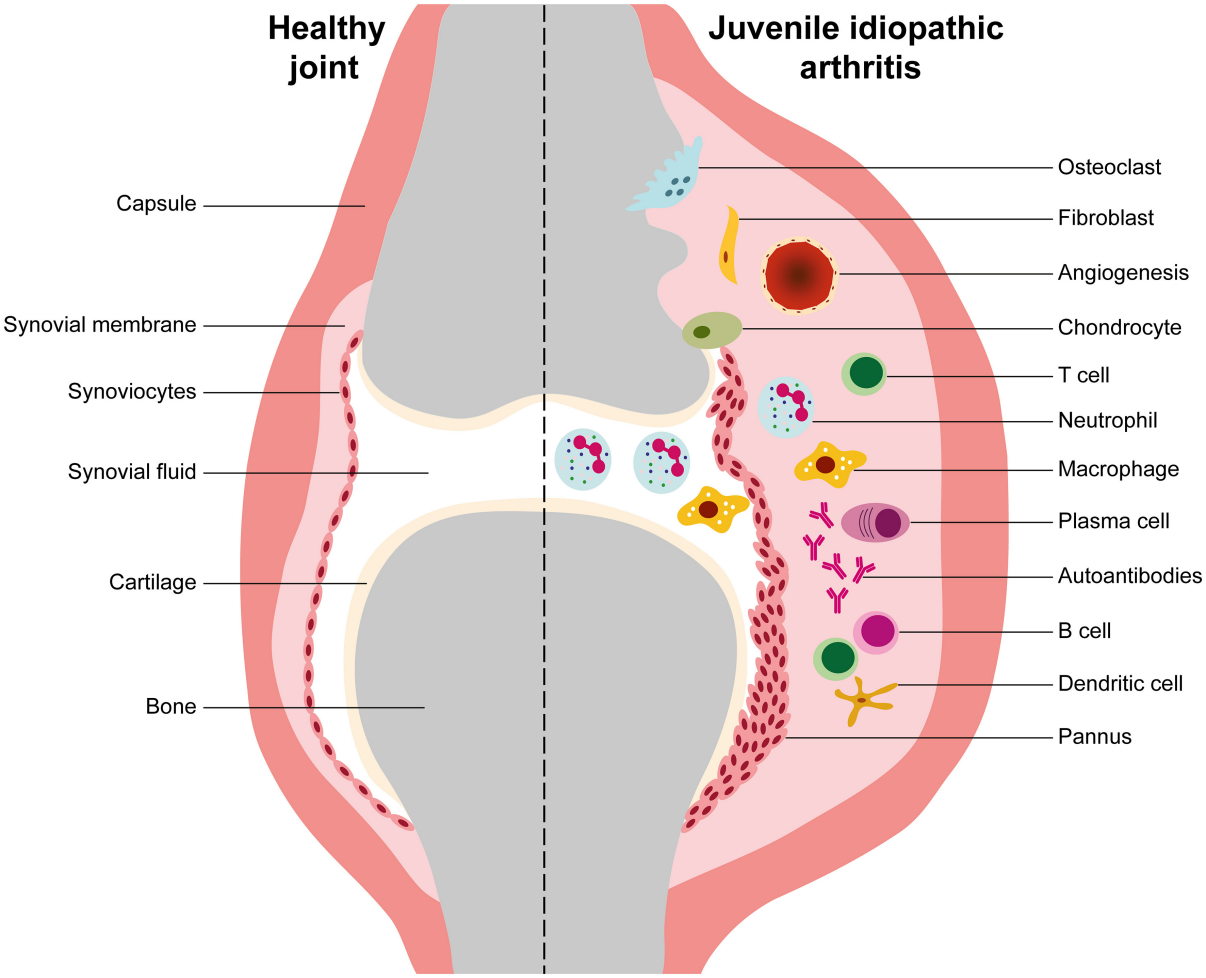
Synovial inflammation in juvenile idiopathic arthritis. This is a representative scheme illustrating the differences between healthy and juvenile idiopathic arthritis (JIA) joints. In JIA, the inflammatory process that occurs at the synovial joint is characterized by an uncontrolled proliferation of synoviocytes and cellular infiltration of the sub-lining layer of the synovium by macrophages, granulocytes, B cells, T cells, and plasma cells, which causes synovial membrane hyperplasia. Consequently, intra-articular hypoxia occurs and pathological angiogenesis initiates. The new blood vessels that form within the synovium increase blood supply and contribute to the migration of pro-inflammatory cells into the joint, thus forming a pathological synovium known as “pannus.” The complex cellular networks that occur within JIA synovium and the production of inflammatory mediators such as cytokines, chemokines and metalloproteinases stimulate chondrocytes and osteoclasts that trigger cartilage and bone erosion, respectively.

The mechanisms of immunopathogenesis of JIA are still poorly understood. JIA categories are complex, heterogeneous, with different contributions of immune system players and effector cells (24, 25, 58–63). Indeed, several studies have demonstrated a predominance of adaptive immunity in the pathogenesis of oJIA, pJIA, ERA, and JPsA (14, 17, 18, 24, 25, 39, 57, 64–86), whereas innate immune responses are the major contributors to disease development and progression in sJIA (29, 59, 87–98). In fact, oJIA, pJIA, ERA, and JPsA are classified as autoimmune diseases, while sJIA has been proposed as an autoinflammatory disorder (25, 58, 59). In JIA, joint inflammation, swelling and tissue destruction are a hallmark of the disease. The pathophysiology mechanisms associated to JIA development are related to an abnormal activation of immune system cells such as B cells, T cells, natural killer (NK) cells, dendritic cells (DCs), monocytes, neutrophils, plasma cells, and to the production and release of pro-inflammatory mediators (cytokines, chemokines, enzymes such as matrix metalloproteinases, aggrecanases, and cathepsins) that ultimately lead to cartilage and bone destruction and systemic manifestations. The inflammatory process that occurs at the synovial joint leads to the thickening of the synovial membrane due to an excessive proliferation of synoviocytes and infiltration of the sub-lining layer of the synovium by immunocompetent cells (lymphocytes, macrophages, granulocytes, plasma cells…), which causes hyperplasia and hypertrophy of the synovium (Figure 2). Consequently, intra-articular hypoxia occurs and pathological angiogenesis initiates. The new blood vessels that form within the synovium increase blood supply and contribute to the migration of pro-inflammatory cells into the joint, thus forming a pathological synovium known as “pannus.” Overall, the complex cellular networks and the release of inflammatory mediators that occur within JIA synovium stimulate chondrocytes and osteoclasts that trigger cartilage and bone erosion, respectively (Figure 2) (99–107).

## B Cell Roles in Juvenile Idiopathic Arthritis Pathophysiology

**Figure 3:**
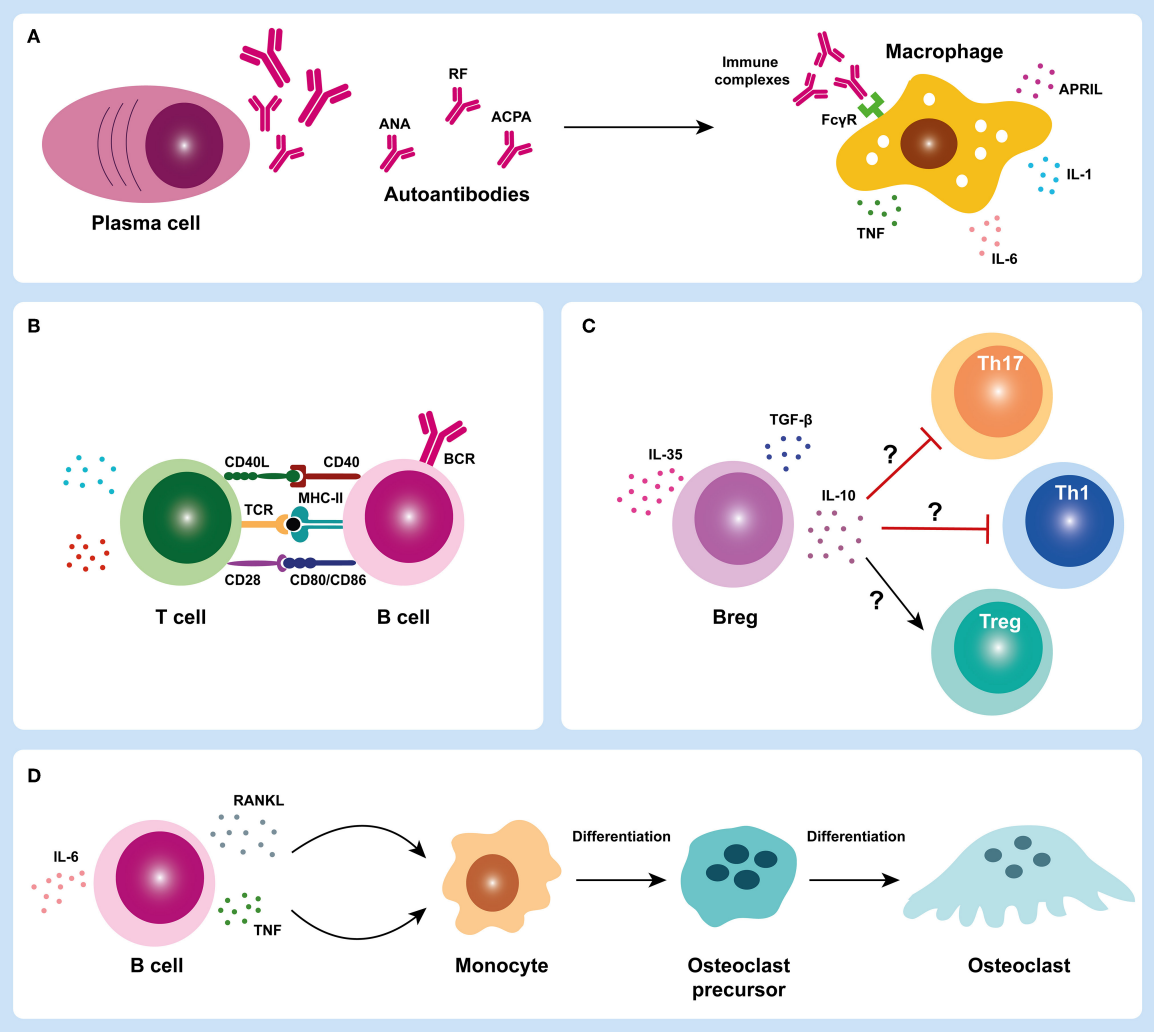
B cell roles in juvenile idiopathic arthritis. B cells can have several important roles in juvenile idiopathic arthritis (JIA) pathogenesis through autoantibody production **(A)**, antigen presentation and/ or T cell activation **(B,C)**, cytokine release and contribute to bone damage in JIA synovial joint **(D)**. In JIA, activated B cells can differentiate into autoantibody-producing plasma cells. Plasma cells produce autoantibodies that can form immune complexes that deposit in the joints and trigger macrophage activation through Fc-gamma receptors (FcγR) **(A)**. Activated macrophages can release several pro-inflammatory mediators such as cytokines (IL-1, IL-6, and TNF) that contribute to the inflammatory process **(A)**. B cells can also act as efficient antigen presenting cells and activate T cells **(B)**. Regulatory B cells (Bregs) may also have an important contribution in JIA pathogenesis through either a defective suppression of T helper cell subsets (Th1 and Th17) and/ or an impairment of regulatory T cells (Tregs) activation **(C)**. B cells can also release cytokines such as TNF and RANKL that can activate osteoclastogenesis and trigger bone erosion in JIA synovial joint **(D)**. ACPA, anti-citrullinated protein antibodies; ANA, antinuclear antibodies; APRIL, A proliferation-inducing ligand; BCR, B cell receptor; Breg, regulatory B cell; CD, cluster of differentiation; FcγR, Fc-gamma receptor; IL, interleukin; MHC-II, major histocompatibility complex class II; RANKL, receptor activator of nuclear factor kappa-B ligand; RF, rheumatoid factor; TCR, T cell receptor; TGF-β, transforming growth factor beta; Th, T helper; TNF, tumor necrosis factor; Treg, regulatory T cell.

JIA has been classically considered a T-cell driven autoimmune disease, except for sJIA subtype, in which innate immune cells have a central role in disease pathogenesis as previously mentioned. However, the detection of autoantibodies reacting with different target antigens in JIA patients suggests a central role of B cells in JIA pathophysiology. In fact, B cells may have important roles in JIA pathogenesis through autoantibody production, antigen presentation, cytokine release, and/ or T cell activation (Figure 3) (5–7). Although the study of B cells has not been extensively explored in JIA, evidence from the literature suggest the occurrence of alterations in B cell differentiation, homeostasis and hyperactivity in JIA patients, which can contribute to disease progression.

### Autoantibody Production

Autoantibody production is a hallmark function of B cells in autoimmunity. In JIA, autoantibodies such as ANA, RF and ACPA can be detected in the serum of these patients (8, 108–114), which supports a breakdown in B cell tolerance. ANA are autoantibodies that can target several autoantigens within cell nucleus structures including nucleic acids, nucleosomes, phospholipids, and several nuclear and nucleolar proteins (115). These autoantigens, which are normally “hidden,” are exposed to antigen presenting cells during cell death, particularly during apoptosis. ANA can be detected in several autoimmune diseases such as systemic lupus erythematosus (SLE), RA, Sjögren's syndrome, idiopathic thrombocytopenic purpura, mixed connective tissue disease, juvenile dermatomyositis, autoimmune hepatitis, primary biliary cirrhosis, ulcerative colitis, and autoimmune thyroiditis (116–125). In JIA patients, the overall seroprevalence of ANA among all subtypes of JIA combined is < 50% (126). ANA are more commonly detected in oJIA and pJIA (mostly in pJIA RF-) patients and are particularly more prevalent in young, female oJIA patients (127). In JPsA patients, ANA positivity is also more strongly associated with early-onset disease and female predominance (39). ANA are less commonly detected in sJIA and undifferentiated JIA (126). Although the exact contribution of ANA to JIA pathology remains unclear, previous reports have suggested that ANA positivity is associated with the development of ectopic lymphoid structures in synovial tissue (16). Interestingly, the presence of a lymphoid organization in synovial tissue from ANA+ JIA patients was strongly related to the concomitant degree of plasma cells infiltration (16). Thus, the development of these lymphoid structures could contribute to the interactions between autoreactive B and T cells, which could directly support the production of these autoantibodies and the inflammatory process in the joint. Furthermore, it has been demonstrated that ANA are associated to a higher risk of uveitis (119, 128). RF is an immunoglobulin of any isotype (predominantly IgM) that specifically recognizes the Fc portion of IgG molecules, which was first described in RA patients (129, 130) and is currently included in the classification criteria for RA (131). RF can be detected in other autoimmune disorders such as SLE, Sjögren's syndrome, systemic sclerosis, mixed connective tissue disease, polymyositis, dermatomyositis, as well as in healthy individuals (132, 133). RF have also been identified in JIA patients (8, 134) and, despite being present in a small subgroup of pJIA patients (only 5% of total JIA patients), RF positivity is associated with a worse disease prognosis (113, 135). Indeed, patients with pJIA RF+ are at higher risk of a more aggressive disease course with cartilage and bone erosions than JIA patients without RF (113, 136–138). Notably, pJIA RF+ is considered the pediatric version of adult RA (139, 140). In fact, it has been demonstrated that the majority of pJIA patients, particularly pJIA RF+, evolve to RA in adulthood (21). Of note, these observations reinforce that pJIA RF+ and RA are likely to have similar underlying pathological mechanisms and that current treatment strategies applied in RA are directly relevant to pJIA RF+. RF can have important physiological roles in the normal immune system such as promoting phagocytosis and the removal of antigen-antibody complexes in the course of infection; fixation of complement; and enhancing B cell antigen uptake and presentation to CD4+ T cells (141). Nevertheless, these naturally-occurring RF are generally low-affinity and polyreactive, whereas pathogenic RF tend to have undergone affinity maturation (133, 142). Although the mechanisms of RF production are not entirely understood, previous reports have described to be dependent on immune-complex recognition by B cell receptors in the context of toll-like receptor stimulation, as well as T cell help (143–145). ACPA are autoantibodies that recognize citrullinated peptides and proteins and, similarly to RF, are included in the classification criteria for RA (131). Although ACPA are highly specific to RA diagnosis, these antibodies can also be detected in JIA patients, particularly in pJIA RF+ (111, 112, 114, 146–148). In fact, it has been demonstrated that ACPA detected in pJIA RF+ patients express the inherently autoreactive 9G4 idiotope, which supports an activation of autoreactive 9G4+ B cells in JIA (148), similarly to what has been described in RA patients (149). Importantly, ACPA detection in JIA patients is associated to more severe and erosive disease progression, which might implicate an earlier and more intensive treatment (8, 112–114, 146, 150, 151). During inflammation, ACPA might be produced in a process called citrullination, a post-translational modification catalyzed by peptidylarginine deiminases (PAD) enzymes in which arginine amino acid residues are enzymatically converted into citrulline residues in a wide range of proteins (152, 153). These structural changes in proteins can form new epitopes that trigger ACPA production by autoreactive B cells. Of note, both ACPA and RF autoantibodies are able to form immune complexes that deposit in the joints. These immune complexes can activate complement and macrophages through Fc-gamma receptors (FcγR) and induce cytokine release that contribute to the inflammatory process in JIA (Figure 3A) (8, 154, 155). Importantly, evidence of hypergammaglobulinaemia correlated with clinical disease activity has been described in JIA patients (mainly in oJIA and pJIA), which is consistent with B cell hyperactivity (14).

### Antigen Presentation, Cytokine Release, and T Cell Activation

During the inflammatory process in JIA, B cells can function as efficient antigen presenting cells and, once activated, can release cytokines and stimulate T cell activation, thus contributing to the exacerbation of inflammation (Figure 3B). A distinctive feature of chronic inflammatory arthritis is the presence of synovial lymphocytic infiltrates that play a role in disease pathogenesis by secretion of pro-inflammatory cytokines and other soluble mediators (156–166). Indeed, both B and T cells are detected in synovial infiltrates from JIA and RA patients (17, 18, 69, 79, 156, 163–165, 167–172). In particular, high levels of plasma cells infiltration have been detected in the synovial membrane of JIA patients (69, 173). Furthermore, it has been shown that lymphoid neogenesis in JIA is correlated to ANA positivity and plasma cells infiltration not only in synovia, but also in iris tissue, which further supports a dysregulated B cell activation in JIA patients (16, 174, 175). Notably, it was shown that JIA patients with ANA+ anterior uveitis often show an infiltrate of plasma cells in iris (174). In addition, it has been demonstrated that activated memory B cells accumulate in the inflamed joints of patients with JIA (17, 18, 171, 176). Of note, it was shown that class-switched CD27+ and CD27- memory B cells expressed up-regulated levels of the costimulatory molecules CD80 and CD86 and could activate allogenic CD4+ T cells *in vitro* more effectively when compared to peripheral blood B cells (18). Additionally, it was observed that synovial B cells could not only induce the activation and polarization of T helper (Th)-1 cells, but also secrete Th1-polarizing cytokines (18). Overall, the accumulation of memory B cells in the synovia of JIA patients suggests antigen-driven activation of B cells within the inflamed tissues potentially triggered by local antigens (18). Changes in B cell subpopulations have also been documented in peripheral blood and synovial fluid from JIA patients (12–14, 17, 18, 92, 177–179). Indeed, expansions of CD5+ B cells and transitional (CD24^high^CD38^high^) B cells have been reported in oJIA and pJIA patients (12–14, 17), which suggest that defects in B cell differentiation and homeostasis may occur in JIA. Of note, CD5+ B cells might be involved in autoantibody production and have the ability to function as antigen presenting cells (180–182). Moreover, it has been demonstrated that transitional B cells (CD24^high^CD38^high^) can secrete interleukin (IL)-10 and regulate CD4+ T cell proliferation and differentiation toward T helper effector cells (183–186). Furthermore, transitional B cells can also secrete pro-inflammatory cytokines such as IL-6 and tumor necrosis factor (TNF) that contribute to disease pathogenesis (187–189). In addition, alterations in regulatory B cells (Bregs) have also been described in JIA patients that might contribute to disease development (178, 190). Indeed, it was shown that the frequency of CD19^+^CD24^high^CD38^high^ Bregs was significantly decreased in peripheral blood and synovial fluid from JIA patients (178). Interestingly, patients with pJIA RF+ had reduced levels of CD19^+^CD24^high^CD38^high^ Bregs when compared to patients with pJIA RF- (178). Moreover, the frequency of IL-10-producing Bregs (B10 cells) was significantly lower in active JIA patients in comparison to inactive patients (178). In fact, alterations in regulatory B cell numbers and/ or functions have been described in several autoimmune diseases (183, 191–195). Previous studies have shown that RA patients have decreased frequencies of CD19^+^CD24^high^CD38^high^ Bregs in circulation and that these cells fail to suppress Th17 cells differentiation (183). Furthermore, RA patients with active disease have reduced levels of CD19^+^CD24^high^CD38^high^ Bregs in peripheral blood when compared to patients with inactive disease, similarly to what has been described in JIA (183). Taken together, these observations suggest that patients with JIA might have altered regulatory B cell functions, with defective suppression of T helper cell subsets (Th1 and Th17) and/ or impaired regulatory T cells activation, as previously described in other autoimmune diseases (Figure 3C) (183, 191–195). In addition, increased levels of cytokines relevant for B cell activation, maturation, differentiation and survival such as B cell activating factor (BAFF), A proliferation-inducing ligand (APRIL), IL-6 or IL-21, have been detected in serum and/ or synovial fluid from JIA patients (76, 89, 196–201). Of note, BAFF and APRIL serum levels from JIA patients were significantly correlated with disease activity (199). Furthermore, elevated peripheral blood BAFF mRNA levels have been described in JIA patients (202). Also, increased levels of IL-6 and IL-21, cytokines relevant for B cell maturation and plasma cell differentiation, respectively, have been found in synovial fluid from JIA patients (76). Interestingly, IL-21 synovial fluid concentration was particularly increased in pJIA patients (76). Moreover, it was observed that a worse disease severity at baseline in JIA patients was associated with increased IL-6 plasma levels (201). Overall, these observations support the occurrence of B cell triggering mechanisms in JIA that contribute to disease progression. B cells can also act as major producers of receptor activator of nuclear factor kappa-B ligand (RANKL), a key cytokine in osteoclastogenesis, and bone erosion (167, 169, 203). Interestingly, it has been shown that JIA patients have significantly increased levels of RANKL not only in serum, but also in synovial fluid (99, 204–206). Importantly, higher levels of RANKL were associated with a more serious disease, particularly in pJIA patients (99, 205, 206). Thus, these observations suggest that B cells might have a critical role in bone damage and joint destruction in JIA (Figure 3D).

## Treatment and B Cell Targeted Therapies in Juvenile Idiopathic Arthritis

Treatment of JIA is adjusted according to the severity of the disease as combinations of non-steroidal anti-inflammatory drugs (NSAIDs), synthetic and/ or biological disease modifying anti-rheumatic drugs (DMARDs) (207–210). Intra-articular corticosteroid injections can also be used with great effectiveness (211, 212). Systemic administration of corticosteroids can have a positive short-term effect, but its prolonged administration is associated with severe side effects such as osteoporosis, growth suppression or immunosuppression (213–215). The American College of Rheumatology (ACR) recommends early use of DMARDs in JIA patients, specifically methotrexate (MTX) (214, 216–218). Furthermore, biological drugs have also been approved for JIA treatment, including TNF inhibitors (etanercept, infliximab, adalimumab, and golimumab) (219–223); the T-cell modulator abatacept (224, 225); the humanized monoclonal antibody against the IL-6 receptor (IL-6R) tocilizumab (226–228) and the IL-1 inhibitor canakinumab (for sJIA) (229–231). Moreover, IL-1R antagonist anakinra (232, 233) and IL-1 inhibitor rilonacept (234, 235) can also be used as effective treatments in sJIA. Additionally, the Janus kinase inhibitor (JAKi) tofacitinib is already approved for the treatment of JIA and clinical trials are currently ongoing to assess the effectiveness and safety of baricitinib in JIA treatment, with preliminary data showing promising results (236–240). Despite the progress achieved in the last years in JIA treatment, about half of the patients continue to require active treatment into adult life, whereas complete remission is reached in only 20–25% of patients (241, 242). B cell depletion therapy with rituximab, a monoclonal antibody that targets CD20 expressed on B cells, is a successful treatment in autoimmune diseases such as RA (243, 244). Nevertheless, few studies have investigated the effectiveness and safety of this treatment option in JIA (7, 245–253). Rituximab is currently only considered in JIA patients refractory to first-line treatments such as TNF inhibitors and standard immunosuppressive therapies, namely MTX (7, 25, 248, 253). Indeed, it has been demonstrated that rituximab is an effective therapeutic option in patients with severe forms of oligoarticular, polyarticular, and systemic JIA, refractory to several prior agents (245–248, 251–253). Adverse events such as infusion reactions, hypogammaglobulinaemia and infections have been reported in pediatric patients after B cell depletion therapy and must be taken into account (254, 255). Nonetheless, rituximab has been reported as an effective and well-tolerated treatment in children, with a low rate of serious infections described in JIA patients, although it is not formally approved by the European Medicines Agency (EMA) for this indication (253). Notably, the efficacy of rituximab treatment in JIA patients strongly supports that B cells play an important role in JIA pathogenesis.

## Conclusions

JIA is the most common rheumatic disorder in children, classified in seven different categories according to ILAR criteria. JIA can cause significant disability and loss of quality of life, if not treated. Disturbances in innate and adaptive immune responses have been implicated in JIA development. B cells are important players in autoimmune diseases and may have roles in JIA pathogenesis through autoantibody production, antigen presentation, cytokine release, and/ or T cell activation. The study of B cells has not been extensively explored in JIA, but evidence from the literature suggests that B cells might have indeed a relevant role in JIA pathophysiology. In fact, the detection of autoantibodies such as ANA, RF and ACPA in JIA patients supports a breakdown in B cell tolerance. Furthermore, altered B cell homeostasis, B cell differentiation, and B cell hyperactivity have been described in JIA, which further supports B cell intervention in disease development. B cell depletion therapy with rituximab is a treatment option considered in JIA patients refractory to first-line biologic treatments such as TNF inhibitors, which has been shown to be an effective and well-tolerated treatment in children with JIA, supporting B cell involvement in JIA pathogenesis. Therefore, further research studies concerning the role of B cells in JIA pathophysiology should be explored, which might be relevant for a better knowledge of disease pathogenesis and have important implications in current and future B-cell targeted therapeutic approaches in JIA.

## Author Contributions

RAM conceptualized the manuscript, reviewed the literature, and wrote the manuscript. JEF revised the manuscript and contributed with important intellectual input. All authors read and approved the final manuscript.

## Funding

The authors would like to acknowledge Sociedade Portuguesa de Reumatologia (SPR) for funding. The funders had no role in study design, data collection and analysis, decision to publish, or preparation of the manuscript.

## Conflict of Interest

The authors declare that the research was conducted in the absence of any commercial or financial relationships that could be construed as a potential conflict of interest.

## Publisher's Note

All claims expressed in this article are solely those of the authors and do not necessarily represent those of their affiliated organizations, or those of the publisher, the editors and the reviewers. Any product that may be evaluated in this article, or claim that may be made by its manufacturer, is not guaranteed or endorsed by the publisher.
